# The role of hypoxic microenvironment in rheumatoid arthritis

**DOI:** 10.3389/fimmu.2025.1633406

**Published:** 2025-08-18

**Authors:** Qiu-han Zheng, Ye Zhai, Ying-hang Wang, Zhi Pan

**Affiliations:** ^1^ Jilin Ginseng Academy, Changchun University of Chinese Medicine, Changchun, China; ^2^ College of Traditional Chinese Medicine, Changchun University of Chinese Medicine, Changchun, China; ^3^ The Affiliated Hospital to Changchun University of Chinese Medicine, Changchun, China

**Keywords:** hypoxic microenvironment, rheumatoid arthritis, hypoxia inducible factor, molecular mechanism, treatment strategy

## Abstract

Rheumatoid Arthritis (RA) is an autoimmune disease caused by many factors, with a high disability rate, unsatisfactory clinical treatment effect, and unclear pathogenesis. The oxygen level in the joint cavity is significantly reduced, and the hypoxic microenvironment has become a key factor in the pathogenesis and progression of RA. Based on the latest research developments, this review delves into the structure and main functions of the key factor HIF in the hypoxic microenvironment, and expounds the main regulatory mechanisms of HIF. The effect of the hypoxic microenvironment on the pathological changes of RA was analyzed, especially how hypoxia affects the signal transduction of related molecules and cells, thus aggravating the occurrence and development of RA. In addition, the review also discusses emerging therapeutic strategies aimed at targeting the hypoxic pathways, including HIF-1α inhibitors, Hyperbaric oxygen therapy, and the application of traditional Chinese medicine. By providing a comprehensive overview of the interplay between RA and the hypoxic microenvironment, this review aims to provide new perspectives on the underlying mechanisms of RA and provide a theoretical basis for the development of therapeutic drugs to improve the hypoxic microenvironment of RA.

## 1 Introduction

**Figure 1:**
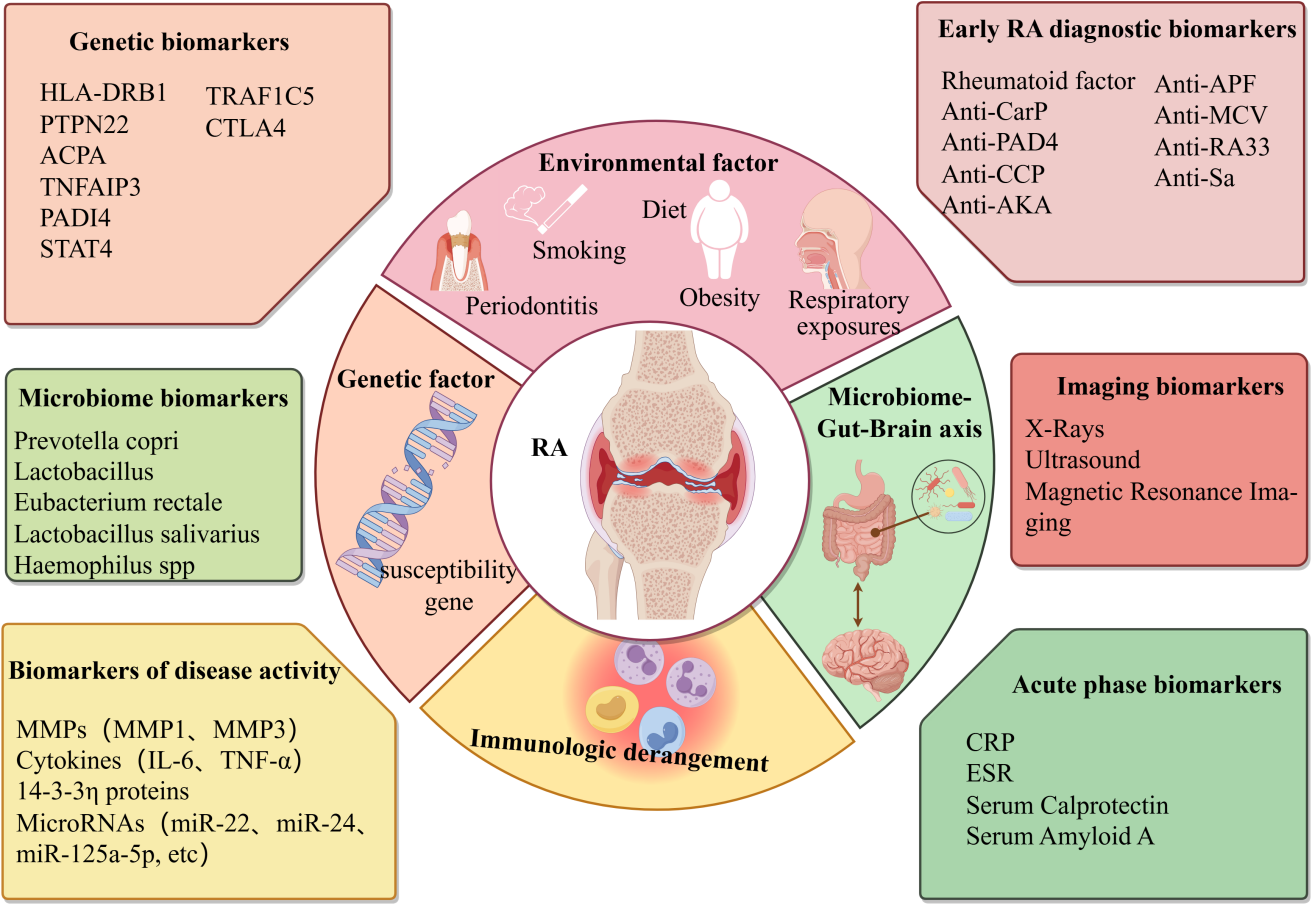
Triggering factors and biomarkers in the pathogenesis of rheumatoid arthritis. Created with figdraw.com.

Rheumatoid arthritis (RA) is a chronic systemic autoimmune disease characterized by synovitis and destructive arthropathy, affecting approximately 1% of the population worldwide (1). The pathological changes of RA are chronic inflammation and abnormal hyperplasia of synovial tissue in the joints, which promote the pannus formation around the joints and then erode the articular cartilage, bone, and surrounding tissues, eventually leading to joint injury, deformity, dysfunction, and even permanent disability (2). According to WHO statistics, with the progression of the disease, RA patients not only experience joint deformity and limited activity but also have an impact on their heart, lung, and nervous system, significantly increasing the risk of cardiovascular disease, osteoporosis, and infection, and seriously affect the quality of life of patients (3, 4). At present, the pathogenesis and biomarkers of RA are not completely clear, involving many aspects, including genetic factors and environmental factors, immune disorders and microbiome-gut-brain axis (**Figure 1**). Therefore, it is of great theoretical significance to deeply understand its disease mechanism and improve the pathological links of RA.

**Figure 2:**
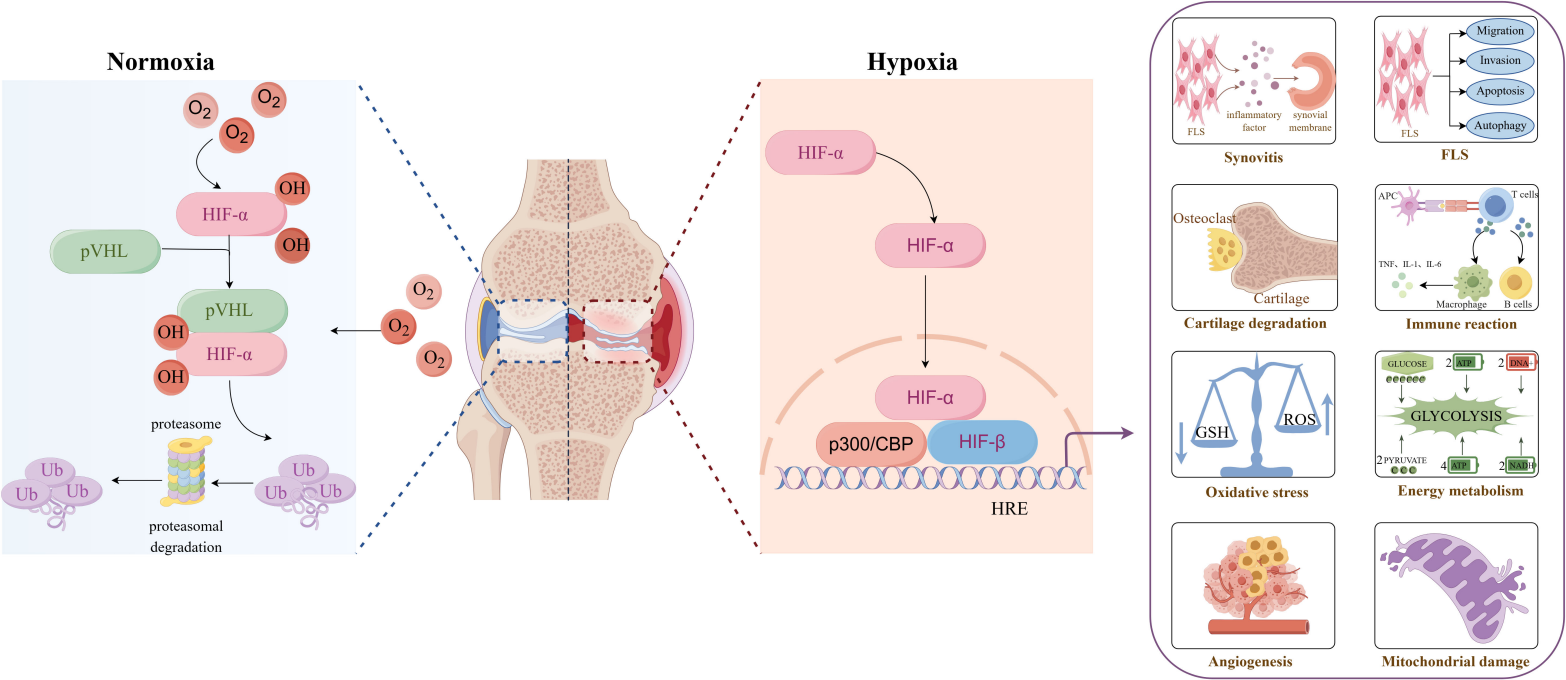
Overview of hypoxic microenvironment on the pathological changes of rheumatoid arthritis. Created with figdraw.com.

Hypoxia refers to the oxygen content in the air, blood and tissues is being lower than the normal level. At present, it has been reported in the literature that compared with the synovial tissue of healthy people, the oxygen partial pressure in the joint cavity of RA patients is significantly reduced. The oxygen partial pressure is only 2-4%, and even less than 1%, suggesting that there is an hypoxic microenvironment in the synovial tissue of RA patients (5). There are two main factors in the formation of hypoxic microenvironment in RA. Firstly, In the pathological state of RA, fibroblast-like synoviocyte (FLS) shows abnormal biological behavior similar to tumor cells, including strong invasiveness and migration ability, excessive proliferation and anti-apoptosis, resulting in active metabolism and decreased blood flow in synovial tissue, resulting in a large increase in oxygen consumption and aggravation of local tissue hypoxia, thus forming an hypoxic microenvironment (6). The second reason is that hypoxia induces FLS to secrete pro-angiogenic factors continuously, promotes the proliferation and remodeling of blood vessels in the synovium, further aggravates local blood circulation disorders, and the oxygen supply mechanism is seriously blocked, eventually causing hypoxia in the joint cavity (7). In 2016, Quiñonez-Flores et al. reviewed the effects of hypoxic microenvironment on RA angiogenesis, inflammatory response, apoptosis, cartilage erosion, abnormal energy metabolism and oxidative damage, and believed that synovial hypoxia is a potential pathogenic factor of RA (8). However, recent studies have found that hypoxic microenvironment can affect mitochondrial function, FLS activity, and the phenotype and function of immune cells by activating HIFs and downstream signaling pathways (9). Based on previous studies, this paper systematically reviews and updates the effects of hypoxic microenvironment on the pathological changes of RA and related molecular mechanisms, and discusses the emerging treatment strategies of hypoxic pathway, aiming to provide a theoretical reference for the in-depth study of the pathogenesis of RA and the development of targeted intervention strategies (**Figure 2**).

## 2 Hypoxia inducible factor in hypoxic microenvironment

### 2.1 Source and structure of HIF

**Figure 3:**
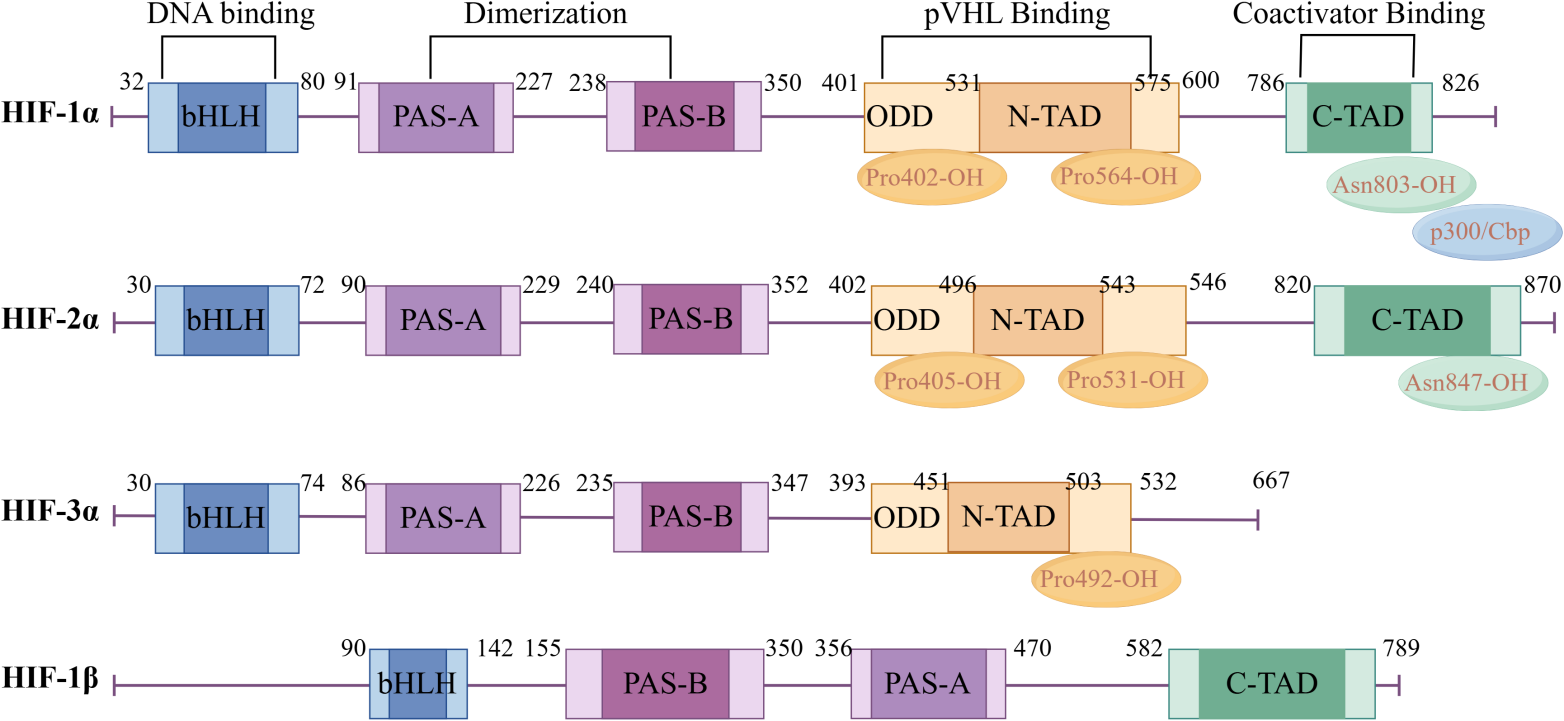
Hypoxia-inducible factor α and β subunit domain structure diagram. Created with figdraw.com.

In 1992, Semenza et al. discovered the transcription factor HIF associated with hypoxic stress under low-oxygen conditions (10). HIF is a heterodimer, which is composed of an N-terminal helix-loop-helix domain (binding to DNA), an intermediate Per-AHR/ARNT-Sim (PAS) domain (promoting heterodimer formation), and a C-terminal transcriptional activation region (binding to transcriptional cofactors and promoting transcriptional regulation). At present, it is found in the human genome that the HIF family is mainly composed of HIF-1,2,3 subtypes and α and β subunits. Among them, the oxygen-insensitive β subunit is constitutively expressed and is stably expressed in cells under normoxic and hypoxic conditions, mainly responsible for the stability of the HIF complex. The oxygen-sensitive α subunit is a functional subunit, and its expression is tightly regulated by cellular oxygen concentration, which is mainly responsible for regulating the activity of HIF. Among the three subtypes of HIF-α, HIF-1α and HIF-2α have great similarities in structure and function (**Figure 3**). They are rapidly stable under hypoxic conditions and induce the transcription of similar target genes (11). However, studies have shown that HIF-1α is activated in strong hypoxia or early hypoxia (<24 hours), while HIF-2α is activated in mild or chronic hypoxia (>24 hours) (12). In addition, differential expression studies have shown that HIF-1α is expressed in all cell types, while HIF-2α is mainly expressed in specific cell types such as endothelial cells, glial cells, and type II lung cells (13). At present, there are few studies on HIF-3α. Some studies suggest that HIF-3α may act as an inhibitory element to regulate HIF-1α and HIF-2α negatively (14, 15).

In the hypoxic microenvironment, HIF dimers can bind to the co-activator p300/CBP (CREB-binding protein) to form a complex, which binds to the promoter region of the target gene containing the hypoxia response element (HRE) to promote its transcriptional expression (16). Although the oxygen level regulates three HIF-α subtypes (HIF-1α, HIF-2α, HIF-3α), and they all form a complex with HIF-1β to recognize and bind to the hypoxia response element HRE with a conserved sequence G/ACGTG upstream of the hypoxia-related gene promoter, HIF-1α is highly sensitive to hypoxia. When the oxygen concentration is lower than 6.0%, the cell HIF-1α level increases exponentially and reaches its highest when the oxygen concentration is 0.5% (equivalent to PO_2_10~15mmHg, 1mmHg =0.133kPa) (17). Therefore, HIF-1α is considered to be the main factor regulating hypoxia response under hypoxic conditions.

### 2.2 Regulation mechanism of HIF expression

HIF-1α is a key regulator of the response of cells to hypoxia in the body, and its expression is regulated in both oxygen-dependent and non-oxygen-dependent ways. Under normoxic conditions, HIF-1α is hydroxylated by prolyl hydroxylase domain proteins (PHDs). The hydroxylated HIF-1α binds to von Hippel-Lindau (VHL) and is rapidly degraded through VHL-mediated ubiquitination modification and the 26S proteasome pathway (18). In addition, the factor inhibiting HIF (FIH) can also specifically modify the Asn803 of HIF-1α to weaken the binding ability of HIF-1α to its transcription cofactor p300/CBP (19). Therefore, HIF-1α is structurally unstable under normoxic conditions, with a half-life of less than 5 min, and its expression is undetectable. Under hypoxic conditions, the activity of PHDs and FIH is inhibited, HIF-1α protein accumulates in the cell, translocates to the nucleus from the cytoplasm, and binds to HIF-1β to form a heterodimer, which binds to the HRE in the promoter region of its target gene to initiate the transcription of related target genes, and participates in various physiological and pathological processes such as erythropoiesis, angiogenesis, autophagy and energy metabolism (20). In addition to oxygen-dependent regulation, the expression of HIF-1α is also affected by non-oxygen-dependent factors such as mechanical stress, hormones, cytokines, growth factors, and low pH. Therefore, HIF-1α, as a core transcription factor in the hypoxic microenvironment, its level continues to rise in RA and plays an important role in the pathological progression of RA (21).

## 3 The mechanism of hypoxic microenvironment in the pathogenesis of RA

### 3.1 Synovitis

Hypoxia is part of the inflammatory microenvironment in RA joints. Under normal circumstances, a dynamic equilibrium state of mutual regulation is maintained between pro-inflammatory and anti-inflammatory factors. When the cells are in a hypoxic state, the expression levels of HIF-1α and HIF-2α in the synovial tissue of RA patients increase, which promotes the secretion of inflammatory factors by FLS, leading to the imbalance of pro-/anti-inflammatory cytokines and aggravates the inflammatory response (22, 23). At the same time, inflammatory factors act on FLS, increase HIF-1α and HIF-2α expression levels, and aggravate tissue hypoxia. A closed loop of self-amplification is formed between the two, resulting in the persistence of RA synovial inflammation. Studies have shown that the expression levels of HIF-1α and HIF-2α are increased in collagen-induced arthritis (CIA) rats. Blocking the HIF pathway by injecting lentiviral vector shRNA plasmid (pLVX-shRNA-conHIF-1α) and HIF-1α gene knockout (lysm-cre/HIF-1α) into CIA rats can reduce the inflammatory response of CIA rat model, reduce the expression levels of TNFα, IL-1β and IL-6 in serum, and reduce the pathological damage of joints, including bone destruction, synovitis and synovial formation (24, 25).

In the hypoxic microenvironment of RA, HIF-1α promotes FLS to produce more IL-33 by activating p38 and acting on the ERK signaling pathway. In addition, IL-33 can reversely induce the expression of HIF-1α in FLS, thereby forming a HIF-1α/IL-33 regulatory circuit and aggravating the inflammation of RA (26). NF-κB is considered the prominent pro-inflammatory family of transcription factors. Under normoxic conditions, activated PHD1 can reduce the expression of NF-κB, thereby inhibiting the transcription of inflammatory factors. Under hypoxic conditions, PHD1 is inactivated, and NF-κB up-regulates the expression of inflammatory factors involved in RA inflammatory response (27, 28). Studies have shown that hypoxia regulates the activity of NF-κB stimulated by classical signaling pathways, and the activation of NF-κB pathway is positively correlated with hypoxia level (29, 30). Under a hypoxic microenvironment, the synergistic effect of the TAK1/NF-κB/HIF-1α signaling pathway was enhanced, and the expression of inflammatory factors IL-6 and IL-8 in FLS cells was induced (31). Hu et al. (32). found that hypoxia and HIF-1α can promote the inflammatory response mediated by the TLR signaling pathway by simulating the hypoxic environment of RA with Na_2_S_2_O_4_. Other studies have shown that HIF-1α can promote the intercellular contact between FLS and T cells and B cells by up-regulating the expression of intercellular contact media and enhancing the secretion of inflammatory factors such as IL-6, IL-8, TNF-α, and IL-1β, thus exacerbating synovial inflammation (33–35). In addition, under hypoxic conditions, HIF-1α can also interact with Notch-3 and STAT-1 to jointly regulate the inflammatory mechanism of RA synovial fibroblasts (36, 37).

**Figure 4:**
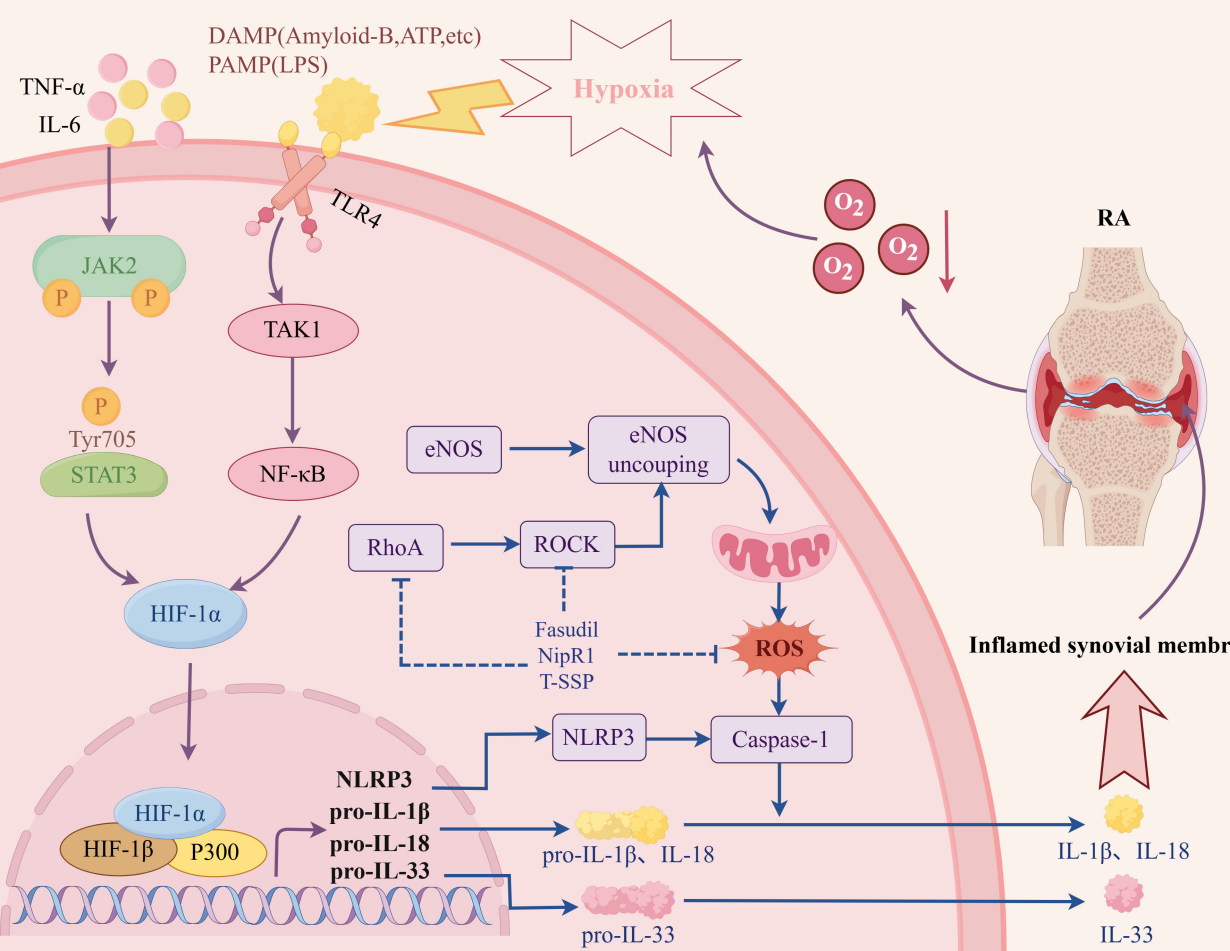
The related mechanism of RA synovitis induced by hypoxic microenvironment. Created with figdraw.com.

In summary, the transcription of related downstream target genes can be initiated under the hypoxic microenvironment through the above-mentioned series of pro-inflammatory signaling pathways (**Figure 4**). The expression of cytokines (IL-1, IL-6, IL-8, IL-15, IL-17, IL-33, TNF-α, IFN-γ), thrombospondin-1, chemokines (CXCL12, CXCL8, CCL20), etc. can be increased, leading to the aggregation of inflammatory cells, further exacerbating the synovial inflammation and other pathological reactions of RA.

### 3.2 Angiogenesis

Angiogenesis, a complex process of new blood vessel formation and expression of multiple genes, is a typical characteristic of RA and is generally connected to hypoxia in both physiological and pathological states. In the hypoxic microenvironment of RA, the continuous accumulation of HIF-1α is a key driving molecule to induce angiogenesis, and the number of HIF-1α positive cells is positively correlated with the infiltration of synovial inflammatory cells and the number of neovascularization (38). Studies have shown that VEGF is a typical hypoxia response gene. When the joint cavity is continuously hypoxic, the activity of PHD is reduced, and HIF-1α and HIF-2α are accumulated in large quantities. The high expression of HIF-1α and HIF-2α increases the expression levels of downstream target genes VEGF and VEGF receptors, induces the release of Angiopoietin-2 from the Weibel-Palade body in vascular endothelial cells, and promotes angiogenesis (39, 40). In addition, VEGF is also a target gene of VHL. Under hypoxia, VHL is disabled, unable to recognize and hydrolyze HIF, resulting in a significant accumulation of HIF, which makes VHL and HIF cooperate, further amplifying the effect, promoting the proliferation and migration of vascular endothelial cells and inducing angiogenesis (41). Notch3 is also a key regulator of pathological angiogenesis. Under hypoxic conditions, Notch3 and HIF-1α synergistically regulate Angiopoietin-2 expression and neovascularization (42). Another study has shown that under hypoxic conditions, the S1P/S1PR1 signaling pathway is activated, and S1PR1 coupled with only inhibited type G protein on EPCs can mediate vasodilation and promote neovascularization (43). Hypoxia can also induce RASFs and SM to increase the expression of MMP, IL-8, and SDF-1 through HIF-1α, degrade extracellular matrix components, and promote angiogenesis (44). Shen K et al. found that Raf/MEK/ERK and PI3K/Akt/mTOR/P70S6K pathways were activated under hypoxic conditions in the articular cavity of RA, regulating the activation of HIF-1α and the transcription and expression of VEGF, and promoting angiogenesis (45). High mobility group protein B1 (HMGB-1) is a non-histone nuclear protein, which belongs to the endogenous TLR ligand. HMGB1 up-regulates HIF-1α transcription through the TLR4/NF-kB pathway, increases VEGF levels, and leads to angiogenesis (46).

**Figure 5:**
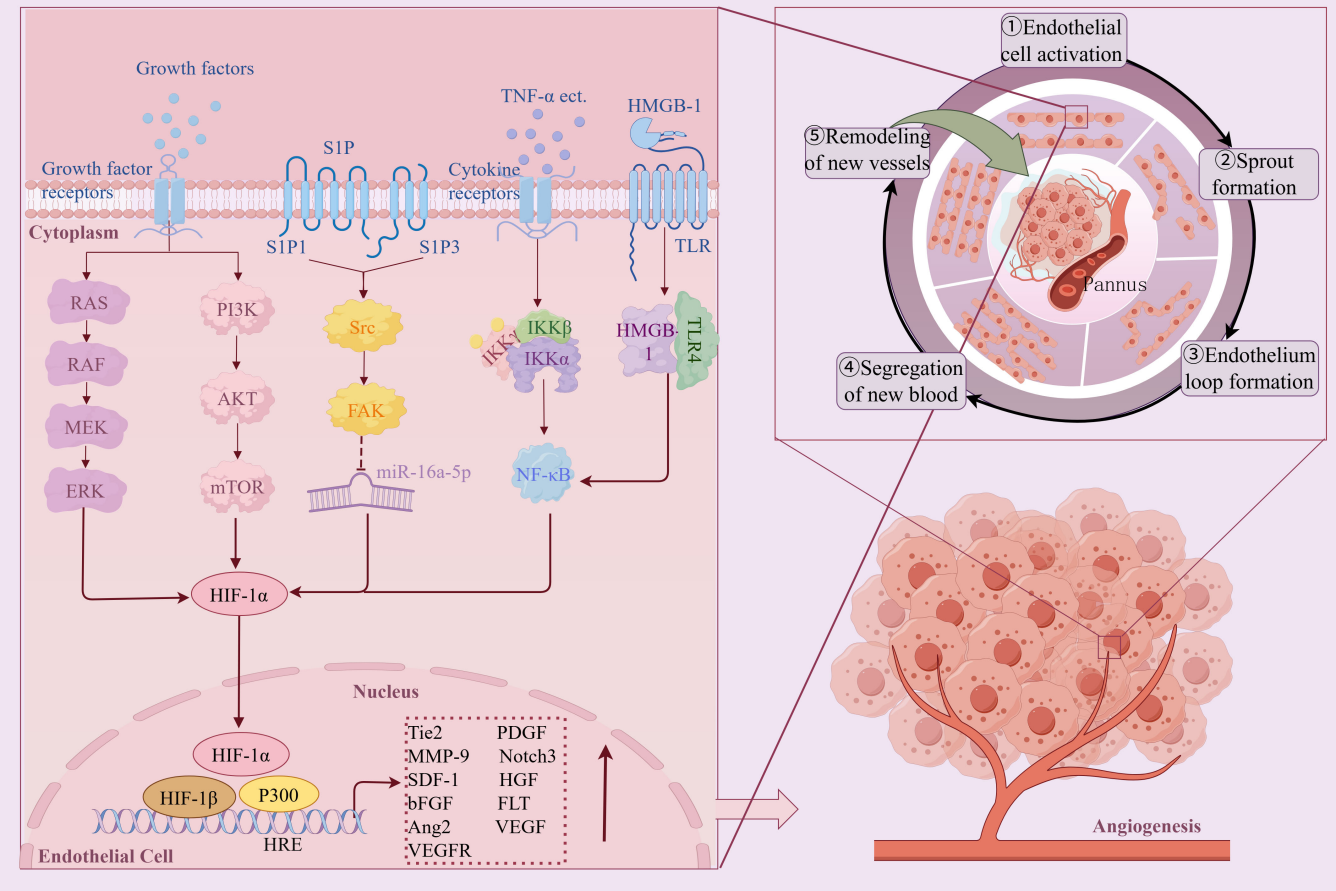
The mechanism and pathway of hypoxic microenvironment induced RA angiogenesis. Created with figdraw.com.

In summary, Angiogenesis, a complex process of new blood-vessel formation as well as expression of multiple genes, is closely related to hypoxia in both physiological and pathological conditions. Hypoxia increases microvascular density in the inflammatory area of synovial tissue by up-regulating pro-angiogenic factors, activating endothelial cells and promoting their migration, while remodeling and degrading extracellular matrix, enabling endothelial cells to invade and form new blood vessels (**Figure 5**). However, the formation rate of new blood vessels is usually unable to catch up with the speed of synovial hyperplasia, and the structure of these new blood vessels is incomplete and cannot effectively provide oxygen. Therefore, despite the formation of new blood vessels, the oxygen supply in the joint cavity is not enough to alleviate the hypoxia of the joint cavity, so that the joint cavity is still in a state of hypoxia, which further aggravates the pathological process of RA.

### 3.3 Cartilage degradation

The destruction of articular cartilage and bone erosion are essential factors for RA dysfunction and disability. The hypoxic microenvironment of RA will lead to an increase in the number of osteoclasts, increased bone resorption, and increased activity of osteolytic enzymes, thereby accelerating bone destruction (47). HIF-1α is a regulator of chondrocyte survival and osteoclast differentiation (48). The continuous hypoxia of the joint cavity can continuously inhibit the activity of PHD. High levels of HIF-1α can directly induce mature osteoclasts to enhance bone resorption and destroy bone and cartilage (49, 50). On the contrary, HIF-1α siRNA can block hypoxia-induced effects (51).

**Figure 6:**
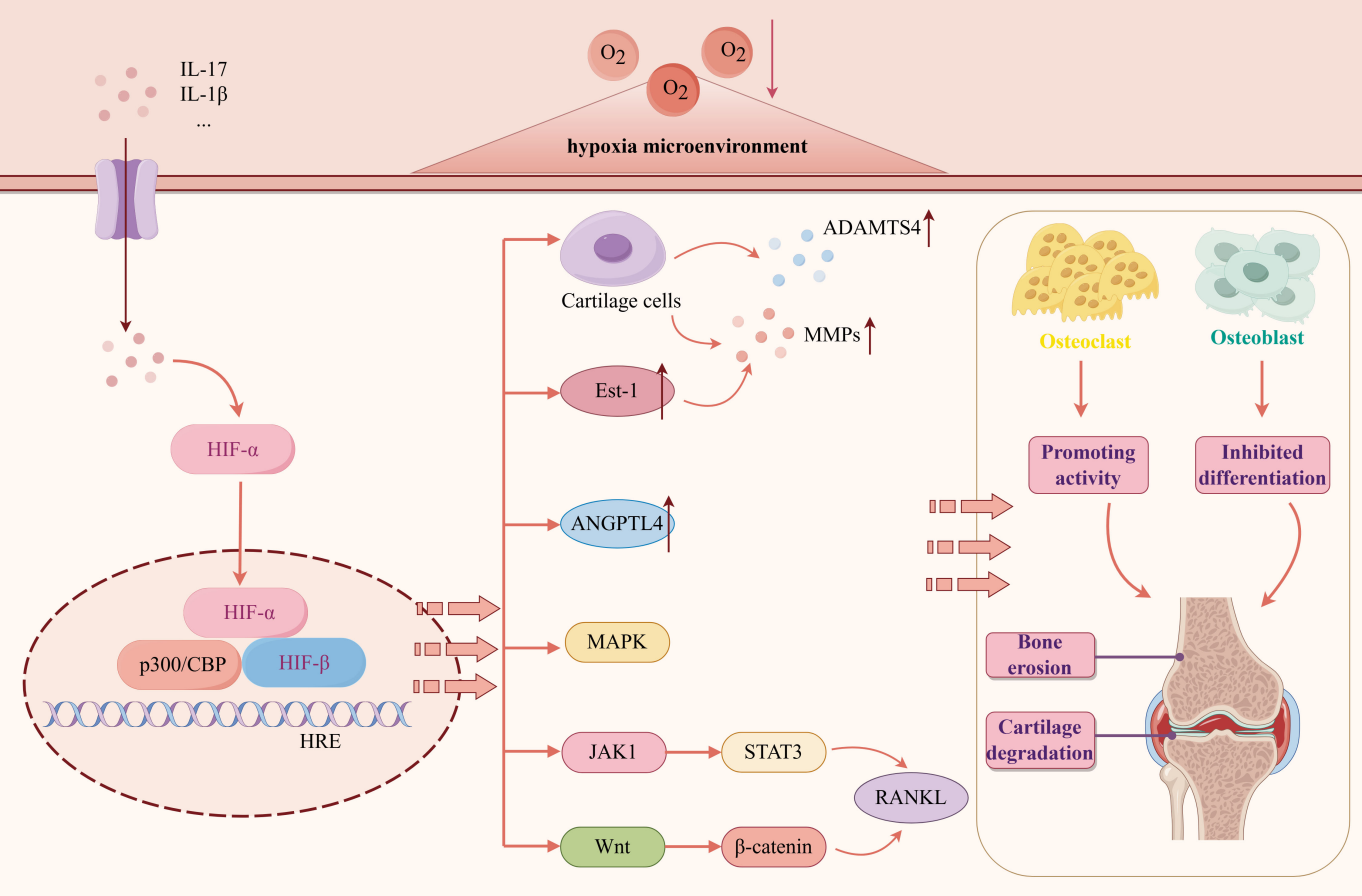
The mechanism and pathway of RA bone destruction induced by hypoxic microenvironment. Created with figdraw.com.

The expression of MMP is increased during tissue damage and remodeling, and the cartilage destruction of the joint is related to the increased activity of MMPs. Under hypoxic conditions, high expression of HIF-1α and HIF-2α can induce chondrocytes to secrete MMPs and a disintegrin and metalloproteinase with thrombospondin motifs-4, promote chondrocyte apoptosis, inhibit chondrocyte autophagy, and aggravate articular cartilage destruction (52, 53). The expression of MMP is induced by Ets-1, which is involved in the invasion and destruction of RA cartilage and bone. The study found that in the AIA rat model, hypoxia can induce the expression of Ets-1 and co-localize with HIF-1α in the synovial inflammatory infiltration site (54). Angiopoietin-like 4 (ANGPTL4) stimulates osteoclast-mediated bone resorption. Under hypoxic conditions in RA, ANGPLT4 is overexpressed in RA osteoclasts in a HIF-1α-dependent manner (55, 56). Highly expressed HIF-1α activates the Wnt/β-catenin signaling pathway, induces excessive proliferation, migration, and adhesion of FLS to cartilage tissue, and ultimately destroys bone and cartilage, resulting in joint deformity (57–60). Other studies have found that hypoxia and proinflammatory cytokines can play a synergistic role in enhancing osteoclast-mediated bone erosion and MMPs expression, thereby aggravating RA bone destruction, mainly through IL-1β/HIF-1α, IL-17/HIF-1α and other pathways (61, 62). RANKL is a central osteoclastogenic molecule. HIF-1α directly increases the osteoclast differentiation of RANKL-mediated RAW264.7 cells *in vitro* by upregulating the MAPK and JAK/STAT pathway under hypoxic conditions in the RA joint cavity (63, 64) (**Figure 6**).

RA bone homeostasis depends on the balance between osteoclast bone resorption and osteoblast bone formation. The above studies reveal that hypoxia can lead to the imbalance between bone resorption and bone formation and promote bone destruction, and HIF plays an essential role in it.

### 3.4 HFLS-RA invasion and metastasis

The tumor-like migration and invasion characteristics of FLS are the basis of pathological processes such as RA bone destruction and invasive pannus formation. In the hypoxic microenvironment of RA, the migration and invasion of FLS are significantly enhanced, resulting in FLS penetrating the vascular wall and eroding articular cartilage and bone, further aggravating joint injury and inflammatory response. Compared with normoxia, the activity and invasiveness of RA-FLS under hypoxic conditions were significantly enhanced, and their invasiveness was positively correlated with the expression level of HIF-1α (65, 66). In addition, high expression of HIF-2α can also promote the proliferation and migration of FLS (67).

In the hypoxic microenvironment, the PI3K signaling pathway is activated, and the activated Akt increases the expression of HIF-1α, MMP-2, and MMP-9 and promotes the epithelial-mesenchymal transition (EMT) of RA-FLS resulting in enhanced migration and invasion (68). This indicates that hypoxia-induced changes in migration and invasion activity of RA-FLS are closely related to the activation of PI3K/Akt/HIF-1α signaling pathway. Fascin-1 can regulate the reconstruction of cytoskeleton and is a key protein to promote cell migration and invasion. In the hypoxic microenvironment, the STAT3 signaling pathway participated in promoting the expression of high accumulation of HIF-1α. Subsequently, the activated HIF-α pathway facilitated the expression of fascin-1, which ultimately contributed to the migration and invasion of FLS (69, 70). Other studies have shown that under hypoxia, IL-17A can up-regulate the expression of MMP-2 and MMP-9 by activating the NF-κB/HIF-1α pathway and promoting the migration and invasion of RA-FLS (71). The hypoxic state of RA can also affect epigenetic factors. Hypoxia-induced ALKBH5 regulates m6A modification of CH25H and aggravates synovial invasion and inflammation in rheumatoid arthritis (72, 73). C-X-C chemokine receptor type 4 (CXCR4) is a vital chemokine receptor widely involved in cell migration and invasion. Under hypoxic conditions, high levels of HIF-1α up-regulate CXCR4 due to the inhibition of PHD2. CXCR4 binds to its ligand SDF-1 and promotes RA-FLS activation, migration, and proliferation (74). The above studies have shown that the hypoxic microenvironment of RA synovium may be an essential reason for the increase of FLS migration and invasion ability.

### 3.5 Mitochondrial dysfunction and cellular stress

Mitochondria, as the ‘ energy factory ‘ of cells, are the main place for aerobic respiration of cells and are very sensitive to changes in oxygen concentration. In the hypoxic microenvironment of RA, mitochondria are mainly characterized by swelling, cristae disappearance, and membrane rupture, causing extensive changes in mitochondrial structure and genomic stability, leading to mitochondrial respiration reduction, oxidative damage, and mitochondrial DNA (mtDNA) mutation accumulation (75–77). Under hypoxic conditions, the expression of mitochondrial marker COX IV in RA-FLS was significantly up-regulated, indicating that hypoxia not only caused mitochondrial damage but also increased the number of damaged mitochondria (78). BNIP3 is a mitochondrial outer membrane protein. The accumulation of HIF-1α under hypoxic conditions promotes BNIP3-mediated mitophagy and NLRP3 inflammasome-mediated pyroptosis, which jointly promote the inflammatory response of FLS (79, 80). Under the condition of joint hypoxia, HIF-1α targets ALKBH7 and down-regulates the expression of UQCRC2 by binding to the ALKBH7 promoter, resulting in mitochondrial damage (81). After mitochondrial damage, the mitochondrial matrix escapes into the cytoplasm, activates cGAS-STING, TLR9, and other signaling pathways, affects the differentiation and function of FLS, OBS, and OCS, destroys the homeostasis of bone metabolism, promotes the secretion of inflammatory factors, and promotes the development of RA (82).

In RA synovial tissue, the persistent hypoxic microenvironment activates cell stress through multiple molecular mechanisms, thereby aggravating the abnormal proliferation of RA-FLS and inhibiting its programmed cell death. Studies have shown that in the hypoxic microenvironment of RA, HIF-1α can inhibit the degradation of wild-type p53 binding protein and increase the expression of p53 by inhibiting the function of Mdm2. However, hypoxia can promote the mutation of p53, which weakens the pro-apoptotic effect of HIF-1α through p53 (83, 84). Another study showed that the level of miR-191-C/EBPβ was negatively correlated with hypoxic stimulation (85, 86). In the hypoxic microenvironment of RA, reducing the expression of miR-191 relieves the inhibition of C/EBPβ, thereby activating its expression and promoting the proliferation of RA-FLS cells while preventing apoptosis (87).

In the early stages of RA disease, FLS can initiate autophagy through various mechanisms, remove denatured proteins and metabolites, and contribute to cell survival (88). However, with the progression of RA disease, with long-term or severe hypoxia, excessive autophagy of FLS triggers autophagy stress, induces damage to cell structure, and then causes continuous damage to cells, leading to RA pathological conditions such as maintaining synovial inflammation, promoting bone destruction, and destroying immune system homeostasis (89). It was found that the expression of autophagy-related proteins p62 and LC-3 in FLS treated with hypoxia was higher than that in normal FLS, indicating that the hypoxic microenvironment in RA joints could induce autophagy in FLS (90). PAD is involved in the post-translational transformation of arginine residues to citrulline, and the expression of citrullinated proteins and antigen presentation induced by PAD are closely related to FLS autophagy (91). In the hypoxic microenvironment of RA, the expression of PAD2 and PADI4 can be increased by regulating HIF-1α, and the production of citrullinated protein can be promoted, thereby inducing FLS autophagy and promoting FLS proliferation (92, 93).

**Figure 7:**
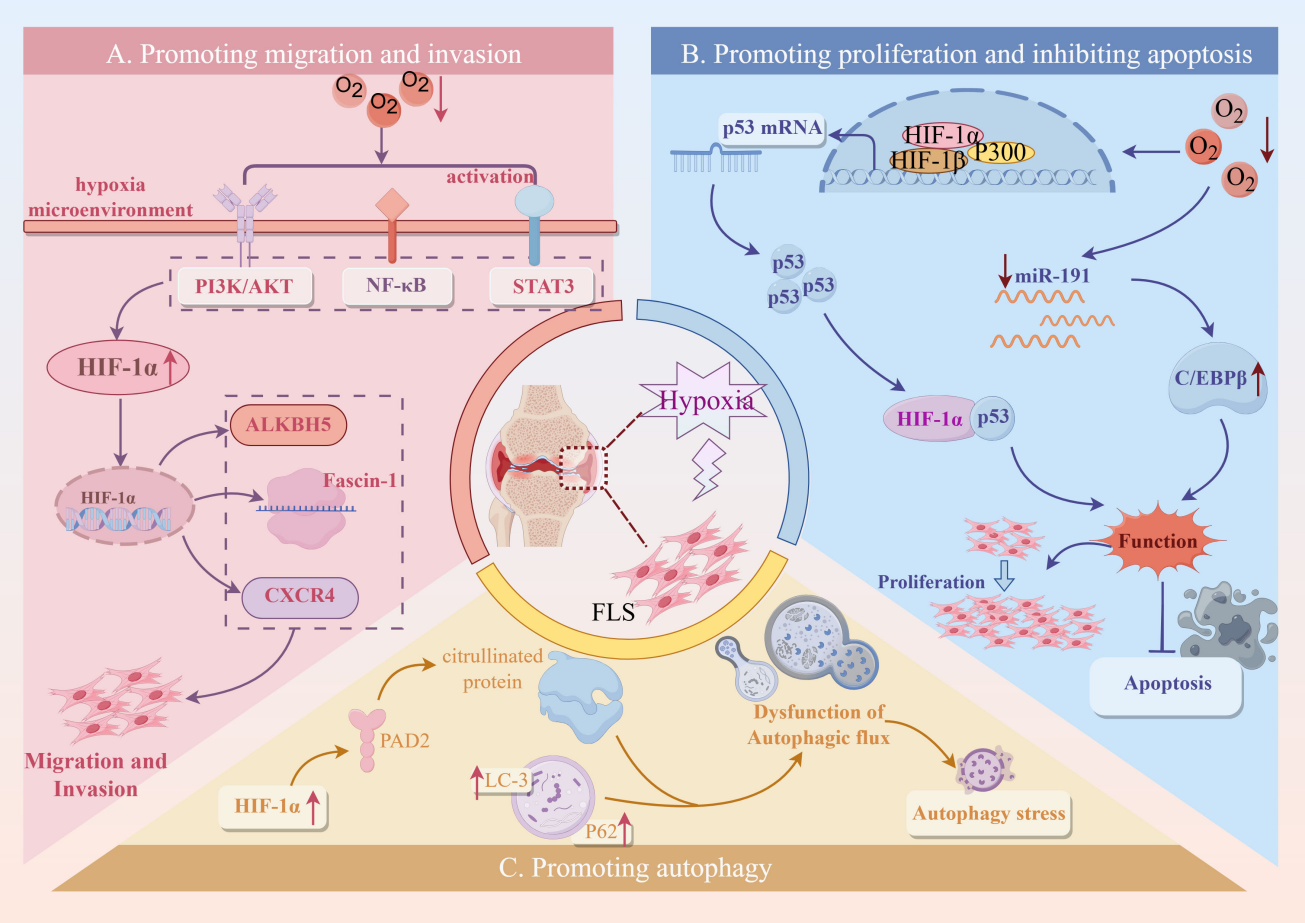
The mechanism and pathway of FLS-RA induced by hypoxic microenvironment. Created with figdraw.com.

In summary, the hypoxic microenvironment in RA synovial tissue induces mitochondrial damage, activates cell stress and autophagy procedures, and affects the survival status and functional behavior of RA-FLS by regulating HIF-1α and its downstream multi-pathways (**Figure 7**). This ‘hypoxia-stress-apoptosis escape and autophagy regulation‘ related response is not only a driving factor for RA chronic inflammation maintenance and bone destruction, but also provides a theoretical basis and potential intervention target for the future development of new therapeutic strategies such as targeting HIF-1α, mitochondrial function protection, and autophagy regulation.

### 3.6 Changes of immune cells

Immune cells are usually the first cells to reach RA lesions and are directly exposed to the stimulation of the hypoxic microenvironment. Hypoxia not only affects the function of innate immune cells (such as macrophages, neutrophils, dendritic cells, and natural killer cells) but also regulates the differentiation of adaptive immune cells (such as T cells and B cells), which promotes the inflammatory state of RA disease and affects the pathological process of RA.

Innate immune cells show heterogeneous responses in function and metabolism in the hypoxic microenvironment of RA. Dendritic cells (DCs) are powerful antigen-presenting cells. Hypoxia can activate HIF-1α, regulate downstream p38/MAPK, PI3K/AKT, and other signaling pathways, affect DCs’ maturation and apoptosis, migration ability, glucose metabolism, antigen presentation ability, promote infiltration in synovial fluid and synovial tissue, aggravate immune response, and promote the continuous activity and pathological progress of RA (94, 95). The infiltration of a large number of macrophages in synovial tissue is an early marker of active RA. The hypoxic microenvironment can upregulate inflammatory factors, such as TNF-α, IL-12, and IL-1β, by activating HIF-1α and HIF-2α. At the same time, it regulates the expression of glycolytic enzymes, promotes the metabolic shift to aerobic glycolysis, and enhances its pro-inflammatory polarization ability (96). Neutrophils are the core cells of innate immunity. The hypoxic microenvironment enhances the pro-inflammatory activity of neutrophils. It inhibits their apoptosis, produces ROS, activates proteases of soluble proteins, induces innate and adaptive immune responses, releases neutrophil extracellular traps, mediates gene expression and cell signal transduction, cell metabolism, and ultimately leads to joint damage (97). In the hypoxic microenvironment of RA, HIF-1α leads to abnormal function of natural killer cells (NK) by inducing MICA/B expression, prompting the immune system to attack its healthy cells indiscriminately, causing tissue damage (98, 99). Other studies have shown that NK function is abnormal, and the ability to release IFN-γ is reduced, thereby losing the inhibitory effect on Th17 cells and exacerbating the inflammatory response (100).

In terms of adaptive immunity, HIF-1α, as a metabolic sensor, plays a core regulatory role in CD4^+^ T cell differentiation. Studies have shown that in the hypoxic microenvironment of RA, HIF-1α can promote the development of Th17 cells by activating RORγt expression and inhibiting FoxP3 transcriptional activity, thereby inhibiting the differentiation of Treg cells and significantly biasing the Th17/Treg balance in a pro-inflammatory direction (101). This HIF-1α-mediated lineage bias is critical for the persistent immune activation of RA. In addition, under the hypoxic microenvironment of RA, HIF-1α not only promotes the polarization of B cells to the IL-6 phenotype, enhances the secretion of inflammatory factors, and activates the STAT3 signaling pathway, but also maintains the survival and function of IL-10-regulated B cell subsets by regulating glycolysis and co-transcriptional regulation of STAT3 (102). The results suggest that B cells are not only antibody producers, but also metabolically dependent inflammatory regulatory hubs in the hypoxic microenvironment of RA. Interestingly, these immune cells do not operate in isolation from each other. Under hypoxic conditions, activated B cells can enhance Th17 response through antigen presentation. Th17 cells secrete IL-17 to stimulate FLS to release IL-6 and chemokines, further recruiting neutrophils and macrophages, thus forming a positive feedback inflammatory loop between cells (103). The regulation and mechanism of hypoxia on various immune cells are shown in **Table 1**.

**Table 1:** The regulation and mechanism of hypoxia on various immune cells.

Immune cells		Mechanism		References
innate immune cells	Dendritic cell	Affect the differentiation and activation of DCs	HIF induces caspase-3 activation, which in turn causes PARP cleavage and promotes apoptosis.	(104)
			Hypoxia promotes the differentiation of immature DC into mature DC.	(105)
		Enhance migration ability	cAMP/PKA	(106)
			p38/MAPK	(107)
			HIF-1α/PI3K/AKT	(108)
		Inhibition of antigen presentation ability	Down-regulation of Cluster of differentiation 209 expression	(109, 110)
		Inhibit glycolysis metabolism	HIF-1α/LncRNADpf3, HIF-1α/p38MAPK	(111)
	Macrophages	Energy metabolism	HIF-1α can regulate the expression of glycolytic enzymes and inhibit oxidative phosphorylation.	(112)
		Affect differentiation ability	mTOR/HIF-1α	(113)
		Promote the secretion of inflammatory factors	HIF-1α/2α promoted the expression of inflammatory factors TNF-α, IL-12, IFN-γ and IL-1β in macrophages.	(114, 115)
	Neutrophils	Inhibit apoptosis	HIF/NF-κB	(116)
		Enhances pro-inflammatory activity	production of chemokines, reactive oxygen species, and neutrophil extracellular traps	(117)
	Natural killer cells	enhances the cytotoxicity	HIF-1α/MICA/B	(99)
Adaptive immune cells	T cells	It promotes the development of Th17 cells and inhibits the differentiation of Treg cells.	Activation of RORgt expression and inhibition of FoxP3 transcription	(101)
		Promote Th1 proliferation and differentiation, inhibit Th2 cell polarization.	HIF-1α/STAT3	(118)
			HIF inhibiting CD4+ effector T cell function	(119, 120)
			Increased expression of surface CD44	(121)
	B cells	regulates the development, differentiation, maturation and antibody secretion of B cells.		(122, 123)

Although existing studies have revealed the regulatory mechanisms of hypoxic microenvironment on the function of various immune cells, the systematic understanding of how these mechanisms intertwine in intercellular interactions and drive RA progression is still insufficient. Therefore, an in-depth study of the interweaving of these mechanisms in a hypoxic microenvironment and elucidating the dynamic regulation map of the immune cell network under hypoxic conditions may provide new perspectives and research directions for understanding the occurrence and development of RA.

### 3.7 Energy metabolism

Energy metabolism has become an essential field in the study of RA. A hypoxic environment significantly affects the metabolic pathway of RA synovial tissue. Under hypoxic conditions, chondrocytes exhibit higher glycolytic activity, increasing glucose consumption and lactate production and increasing the expression of glycolytic genes such as LDHA and PGK1. In addition, hypoxia induces RA tissue to switch from oxidative phosphorylation to glycolysis by activating HIF-1α, reprogramming the energy metabolism pathway of cells (124, 125). Further studies have shown that under hypoxic conditions, up-regulation of HIF-1α leads to an increase in glucose transporter GLUT1 and GLUT3 in RA synovial tissue, thereby promoting glucose uptake and further enhancing the process of glycolysis by regulating the activities of hexokinase II, glyceraldehyde 3-phosphate dehydrogenase and mitochondrial cytochrome oxidase (126, 127).

**Figure 8:**
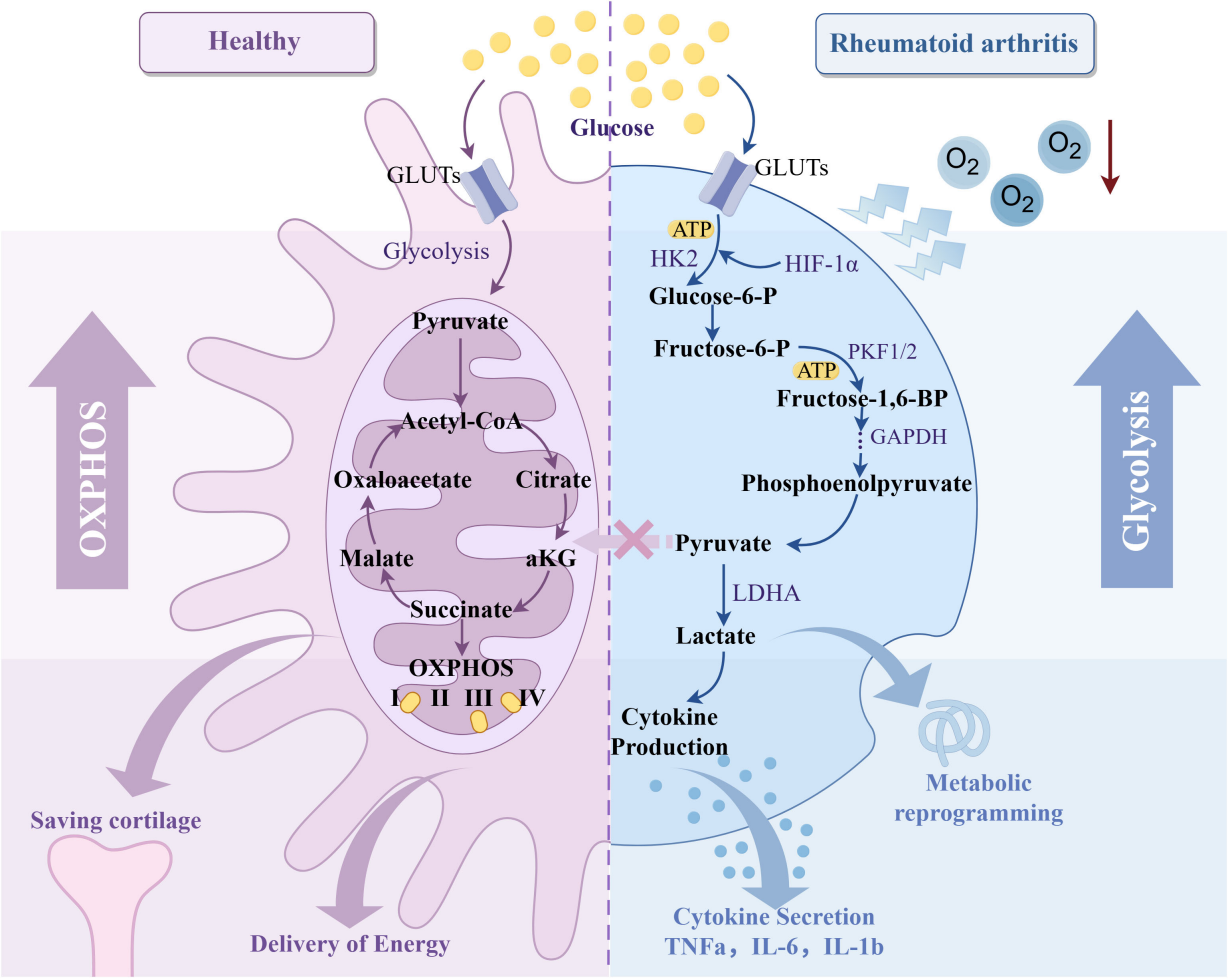
Effect of hypoxic microenvironment on RA glycolysis. Created with figdraw.com.

Based on the above studies, it is found that the direct effect of hypoxia on RA energy metabolism is mainly reflected in two aspects: On the one hand, cells in RA synovium often rely on anaerobic glycolysis to maintain energy supply. This metabolic change not only leads to a decrease in the efficiency of ATP production, which makes cells face energy shortage but also increases the accumulation of local lactic acid, which leads to the formation of a local acidic environment and further aggravates the inflammatory response. On the other hand, hypoxia changes the metabolic pathway of cells by activating HIF, increases glycolysis and glucose uptake, reduces oxidative phosphorylation, and leads to metabolic reprogramming. Long-term hypoxia not only leads to a decrease in the efficiency of energy generation but also further aggravates RA tissue damage (**Figure 8**). Therefore, hypoxia directly affects the energy metabolism of synovial and intra-articular cells in RA patients, aggravating the inflammatory response and promoting the process of joint injury.

### 3.8 Oxidative stress

Under the normal physiological state, the body’s oxidation-antioxidation function maintains a dynamic balance within a specific range. When subjected to hypoxic stimulation, the oxidation system becomes unbalanced, and the oxygen molecules in the cell’s mitochondrial electron transport chain, serving as the final electron acceptor, are insufficient (128). The supply of oxygen molecules leads to the production of a large amount of ROS and accumulation in the cell, which causes damage to the macromolecules and organelles in the cell, activates the inflammatory response, and aggravates the clinical symptoms of RA (129).

Under hypoxic conditions, nitric oxide synthase and NADPH oxidase activity in FLS are upregulated, which promotes the formation of harmful oxidizing substances, such as peroxynitrite, thereby exacerbating inflammation and oxidative damage to joints (130). HIF-1α is the core regulator of the hypoxia response, which can activate redox-related enzymes, such as NADPH oxidase, and increase the production of ROS, thereby inducing oxidative stress (131). Additionally, Nrf2 is a vital antioxidant transcription factor that regulates the expression of antioxidant enzymes, such as superoxide dismutase and glutathione peroxidase, to protect cells from oxidative damage (132). Under hypoxic conditions, the activation of Nrf2 may be inhibited, leading to a decrease in the antioxidant defense system, which in turn exacerbates oxidative stress and inflammatory responses (133, 134). The above studies suggest that hypoxic environment is not only the cause of oxidative stress but also may be a key node in the interaction between oxidative damage and inflammatory response in the pathological process of RA. Therefore, oxidative stress is closely related to hypoxia, and the two work together to promote inflammatory pathways and aggravate disease progression.

## 4 Drugs improving hypoxic microenvironment in the RA

### 4.1 Regulating hypoxia response pathway

#### 4.1.1 HIF inhibitors

HIF is the main transcription factor to adapt to hypoxia, which can regulate the downstream gene spectrum and participate in the pathological process of RA. The existing HIF inhibitors mainly improve the hypoxic microenvironment by reducing the expression of HIF-α protein, inhibiting the transcriptional activity of HIF-α, inhibiting the dimerization of HIF-α and HIF-β, accelerating the degradation of HIF-α, and inhibiting the binding pathway of HIF-α and downstream target genes. It has been found that HIF-2α antagonist PT2399 can change its molecular structure by binding to the PASB domain of HIF-2α, interfere with or inhibit the expression of HIF-2α, and thus play a role in cartilage protection (135). Recent studies have found that HIF-1α inhibitor AMSP-30m can improve the hypoxic microenvironment of RA, inhibit the proliferation of MH7A cells, and improve the symptoms of arthritis in CIA rats by inhibiting Sonic Hedgehog Pathway (136, 137). Other studies have shown that in RA patients with higher CRP levels, the expression of HIF-1α is increased, and HIF-1α competes with AHR to bind to ARNT, which limits the efficacy of Leflunomide. The combination with HIF inhibitor Acriflavine can reduce the binding of ARNT with HIF-1α, promote Leflunomide activating AHR to inhibit CRP production, and inhibit bone erosion in CIA rats with no obvious toxicity. In the future, through in-depth exploration of the pathological mechanisms of RA, the combined application of traditional drugs and targeted agents has led to the development of more targeted therapeutic drugs with a precise mechanism of action. This provides a new direction for RA in precision medicine, combined therapy, and targeted therapy (138). In addition, Other inhibitors of HIF, such as Belzutifan, anti-platelet aggregation agent YC-1, and echinomycin, mainly focus on improving the hypoxic state of tumors (139–141). However, since the hypoxic microenvironment and HIF-1α overexpression in RA synovium are similar to those in solid tumors, these HIF-1α inhibitors are expected to become a new strategy for the treatment of RA.

#### 4.1.2 Prolyl hydroxylase activator

In the hypoxic microenvironment, PHD activity is inhibited, resulting in high expression of HIF-α and participating in pathological processes such as RA inflammatory response, angiogenesis, and bone destruction by affecting downstream target genes. Therefore, by using a PHD activator, the activity of PHD in a hypoxic environment can be increased, and the degradation of HIF-α can be accelerated, thus playing a role in the treatment of RA. It has been found that diacylglycerol kinase inhibitors can activate PHD and inhibit the aggregation of HIF-1/2α protein under high and low oxygen concentrations, thereby inhibiting the activity of HIF-1/2α (142). HJ Choi et al. found a PHD2 activator KRH102053. They proved that KRH102053 can inhibit hypoxia-induced angiogenesis, cell migration and invasion, and glycolysis by measuring the levels of HIF-1α and its downstream target genes (143).

In summary, HIF inhibitors and PHD activity regulate the hypoxic response pathway by directly or indirectly regulating HIF gene expression, and have the potential to improve the hypoxic microenvironment and treat RA and other related diseases. Despite their promise, they are still experimental agents, primarily used in animal and cell studies, and have not yet been officially marketed for clinical treatment of RA. Its clinical transformation faces multiple challenges related to pharmacokinetics and resistance mechanisms, and further research and verification are urgently needed.

### 4.2 Improving tissue oxygen supply

Hyperbaric oxygen therapy (HBOT) refers to the method of inhaling pure oxygen to treat diseases under more than one atmospheric pressure, which can provide more O2 for damaged tissues, improve the physiological function of tissues and accelerate the recovery of tissues (144). At present, HBOT has been used as a treatment for arthritis. It can increase the oxygen transport and uptake of tissues, significantly reduce the expression of HIF-1α, and improve the hypoxic microenvironment of joints (145). Dulberger et al. confirmed the effectiveness of HBOT in the treatment of RA through the standard method of MRI quantification of inflammation and injury (146). Studies have found that HBOT can improve the common pathological angiogenesis of RA, and its mechanism is mainly through promoting the expression of PHD2, accelerating the degradation of HIF-1α, and inhibiting the production of angiogenic factor VEGFA (147). In addition, HBOT can also effectively increase the oxygenation of RA tissues, reduce inflammatory cell infiltration and inhibit the production of inflammatory factors (148). HBOT can inhibit HMGB-1/RAGE signaling pathway in chondrocytes and restore cartilage damage (149). HBOT can promote the transformation of Th17 cells to Treg cells in RA mouse model, which helps to restore the balance of the immune system and reduce the immune injury of RA (150). However, clinical studies have found that the benefits of HBOT are not permanent, and pain symptoms reappear within weeks to months after the last HBOT (151). Therefore, HBOT can be used as an adjuvant therapy, combined with other drugs for the treatment of RA as a new treatment method.

### 4.3 Blood-activating, stasis-resolving and collateral-dredging method

RA belongs to the ‘arthralgia syndrome’ category in traditional Chinese medicine. Traditional Chinese medicine believes that RA is closely related to the disorder of qi and blood and the obstruction of collaterals. When qi and blood are not running well, local tissues do not get enough oxygen and nutrition, resulting in increased hypoxia and inflammatory response in joint tissues. Therefore, traditional Chinese medicine, with the effects of promoting blood circulation, removing blood stasis, and dredging meridians and collaterals, can be used to restore the qi and blood flow of RA, inhibit the proliferation of vascular smooth muscle cells and endothelial cells, thereby inhibiting vascular proliferation and neovascularization, improving the blood supply of joints, and alleviating local hypoxia (152, 153). Salvianic acid A, the active ingredient of traditional Chinese medicine, can down-regulate the expression of inflammatory factors by inhibiting NF-κB/HIF-1α and HIF-1α/STAT3/NLRP3 signaling pathways, inhibit cell excessive proliferation, and improve hypoxia-induced FLS injury (154). Geniposide restored the abnormal sphingolipid metabolism induced by the FLS hypoxic microenvironment (155). Salidroside can effectively alleviate the hypoxic state of CIA model mice and alleviate the hyperplasia of synovial tissue, angiogenesis, and inflammatory symptoms of RA (156, 157). Curcumin can inactivate HIF-α, down-regulate VEGF expression, and reduce RA angiogenesis and inflammatory response (158, 159). Therefore, some compound Buyang Huanwu decoctions with the effects of Yiqi Huoxue and Tongjing Huoluo can inhibit the BNIP3-PI3K/Akt pathway, thereby inhibiting hypoxia-induced mitophagy in FLS (160). Traditional Chinese medicine has shown certain potential in improving the hypoxic microenvironment of joint tissue in RA patients through its unique efficacy in promoting blood circulation, removing blood stasis, and dredging meridians.

In summary, these therapeutic implications of drugs on RA (**Table 2**), are designed to optimize the hypoxic microenvironment through multifaceted pathways, ultimately curbing inflammation and mitigating tissue damage, thereby achieving the desired therapeutic outcomes for autoimmune diseases. In the future, combining traditional Chinese medicine, components, and compound prescriptions with modern molecular biology technology will further enrich the contemporary connotation of arthralgia, more accurately reveal its mechanism of action, and give full play to the characteristic advantages of traditional Chinese medicine in intervening hypoxic microenvironment.

**Table 2:** Therapeutic strategies targeting hypoxic microenvironment in rheumatoid arthritis.

Drugs		Mechanism	Reference	Structure
HIF-α inhibitors	PT2399	HIF-2α inhibitor, Inhibit heterodimerization	(135)	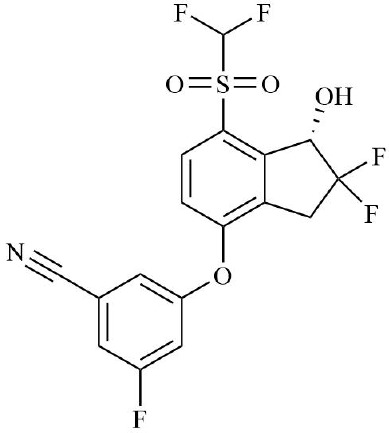
	Belzutifan (MK6482)	HIF-2α inhibitor, Inhibit heterodimerization	(141)	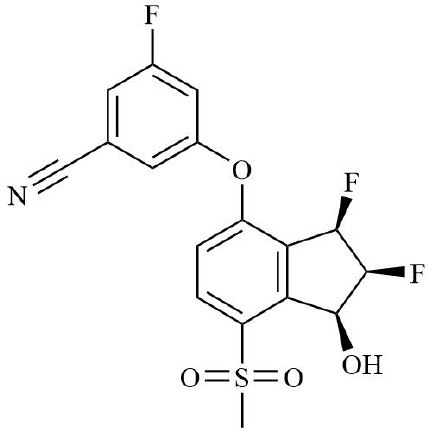
	AMSP-30m	HIF-1α inhibitor, Inhibiting Sonic Hedgehog Pathway	(136, 137)	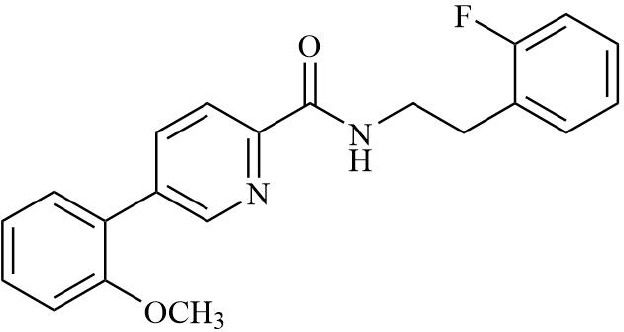
	Acriflavine	HIF-1α inhibitor, facilitate Leflunomide-AHR-CRP signaling, Prevent the dimerization of HIF-1α and HIF-1β subunits	(138)	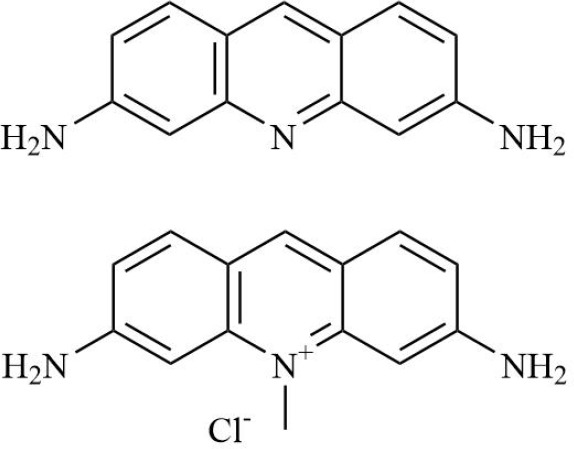
	Lificiguat (YC-1)	HIF-1α inhibitor, Inhibit the transcriptional activity of HIF-α	(139)	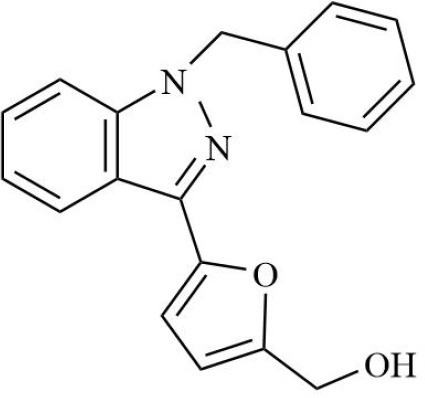
	Echinomycin	HIF-1α inhibitor, Inhibit DNA binding	(140)	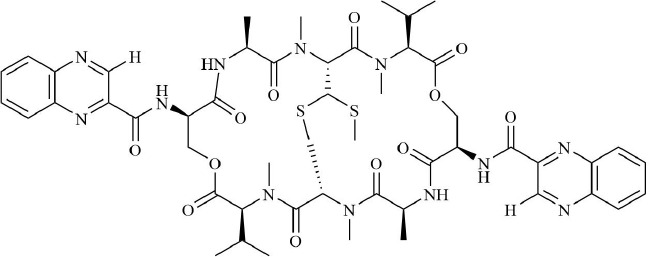
PHD activator	R59949	Inhibition of HIF-1/2α protein aggregation	(142)	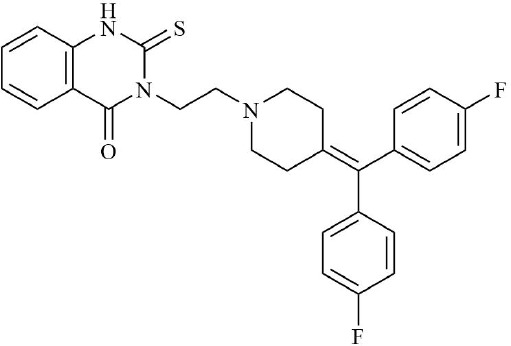
	KRH102053	Inhibition of HIF-1α and its downstream target genes	(143)	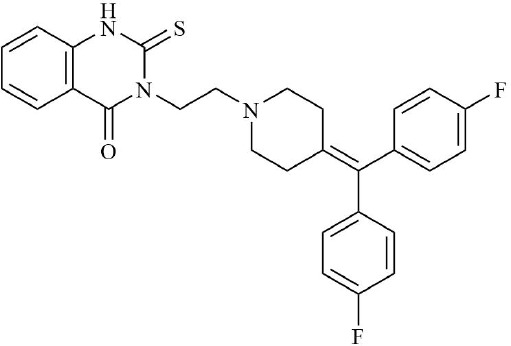
Improve tissue oxygen supply	Hyperbaric oxygen therapy	Regulating PHD2/HIF-1α and inhibiting angiogenesis	(147)	/
		Increase tissue oxygen supply and inhibit inflammatory response	(148)	
		Protects articular cartilage by inhibiting HMGB1/RAGE signaling pathway	(149)	
		Promote the polarization of Th17 to Treg and regulate the immune response.	(150)	
Blood-activating, stasis-resolving and collateral-dredging method	Salvianic acid A	Inhibition of NF-κB/HIF-1α signaling pathway inhibits inflammatory response.	(154)	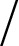
	Geniposide	Inhibition of sphingolipid metabolism	(155)	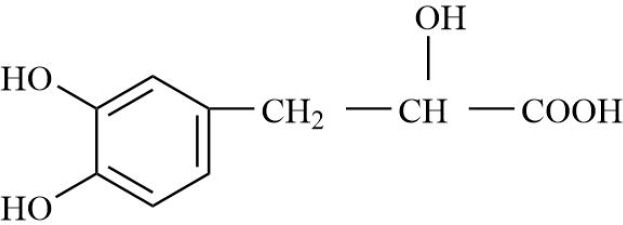
	Salidroside	Relieve synovial hyperplasia	(156, 157)	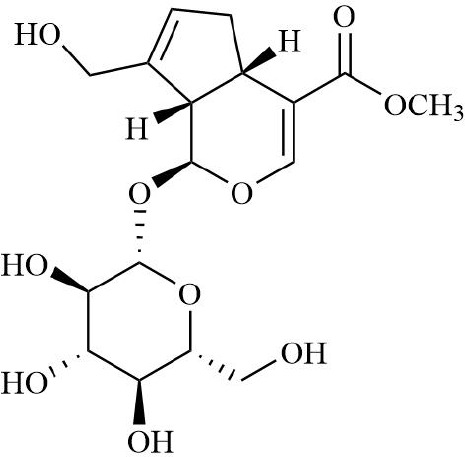
	Curcumin	Inhibition of HIF/VEGF pathway, inhibition of angiogenesis	(158, 159)	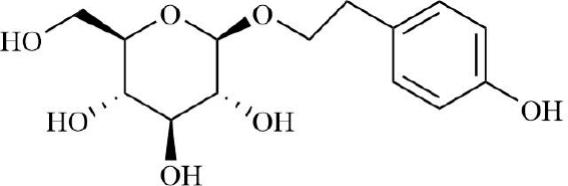

## 5 Discussion

The relationship between hypoxic microenvironment and RA pathological process is a two-way interaction. A large number of proliferating FLS and infiltrating inflammatory cells increased local oxygen consumption (161). At the same time, the proliferation and remodeling of blood vessels in the synovium cause the new blood vessels to extend into the articular cavity, resulting in increased intra-articular pressure and intermittent collapse of the articular capsule capillary network, limiting the effective supply of oxygen (162). The synergistic effect of the above multiple factors leads to long-term hypoxia of synovial tissue and the formation of a RA hypoxic microenvironment. By activating HIFs and downstream signaling pathways, promoting angiogenesis factors, and increasing the recruitment of immune cells, the hypoxic microenvironment itself aggravates the pathological processes of synovitis, angiogenesis, cartilage destruction, mitochondrial damage, cell migration, and invasion of RA. In addition, the hypoxic microenvironment also regulates the differentiation of immune cells, promotes the inflammatory state of RA disease, and interacts with other signaling pathways, thereby promoting the persistence of RA pathology. Therefore, the hypoxic microenvironment is not only the result of the pathological process of RA but also plays a role in promoting the progress of RA.

This article summarizes the literature and finds that HIF, as the main factor regulating hypoxia response under hypoxic conditions, plays multiple roles in different stages of RA. In the stage of RA disease, HIF is usually activated under strong hypoxia or acute hypoxia. As a driving factor, it promotes the secretion of pro-inflammatory cytokines, thus promoting the formation of immune response and inflammatory response and the pathogenesis of RA. In addition, HIF also provides basic conditions for angiogenesis and joint injury. In the progression of RA, HIF is an intermediary factor, which is involved in the destruction of articular cartilage, angiogenesis, and the proliferation and migration of FLS cells by maintaining the hypoxic environment, and promotes the continuous deterioration of the disease. With the extension of the course of RA, the expression of HIF may no longer directly drive the disease, but as a result of pathological reactions, reflecting the persistence of inflammation and cell damage, and enhancing the immune response to promote the recurrence of the disease. Therefore, in the future, we can focus on the mechanism of HIF in different stages of RA, and provide new perspectives and strategies for its targeted therapy.

Currently, the potential drugs to improve the hypoxic microenvironment for treating RA mainly include HIF inhibitors, prolyl hydroxylase catalysts, traditional Chinese medicines, active ingredients, and compounds that promote blood circulation and remove blood stasis. Its mechanism of action is mainly to improve the hypoxic microenvironment by regulating the hypoxic response pathway, improving tissue oxygen supply, and promoting blood circulation to remove blood stasis and dredging collaterals, and then to intervene in various pathological changes of RA, which provides a new strategy for RA treatment.

However, the research on the hypoxic microenvironment of RA is still in its infancy. First, RA is a process of multi-mechanism and multi-signal pathway regulation. There are many cross-effects and influencing factors in multiple pathways regulated by hypoxia-mediated HIF-1α transcription factors. Whether HIF-1α has spatiotemporal dynamics in different links of RA pathological progression, how these signaling pathways coordinate and interact with each other, and the complex relationship with other immune responses or synovial hyperplasia still need further study. Secondly, the potential drugs for improving the hypoxic microenvironment primarily focus on pharmacological inhibitors. However, research on HIF inhibitors at home and abroad has focused mainly on the field of tumors. Its application in RA is still in the preclinical research stage, involving animal experiments or *in vitro* models, and has not been approved for the clinical treatment of RA patients. It is necessary to explore its mechanism of action further and promote clinical transformation. Traditional Chinese medicine and active ingredients can effectively improve the hypoxic microenvironment due to their multi-target and safety advantages, showing certain potential in the treatment of RA. However, at present, it only focuses on a particular pathological link of RA. Still, it ignores the unique advantages of traditional Chinese medicine in improving multiple pathological changes of RA and reducing joint damage of RA.

Therefore, the future will focus on the following aspects. 1) Reveal the hypoxia mechanism of RA tissues and cells: Through single-cell sequencing, proteomics, and gene knockout technology, the interaction mechanism of HIF-1α on downstream signaling pathways and its temporal and spatial dynamic changes in the hypoxic microenvironment of RA was deeply explored, which provided a new perspective and new ideas for the development of new diagnostic biomarkers and the determination of new therapeutic targets. 2) Enhance the targeting efficiency of inhibitors and reduce side effects: Because of the pharmacokinetics of HIF inhibitors, liposomes, and other technologies are used to improve the stability and biocompatibility of HIF inhibitors, and more clinical trials and animal-related studies are carried out to evaluate their efficacy, accurately control the drug dose and time, and reduce the development of side effects and drug resistance. 3) Give full play to the advantages and clinical transformation of traditional Chinese medicine: Based on the thought of syndrome differentiation and treatment of traditional Chinese medicine, classical clinically effective traditional Chinese medicine was selected as the research object, focusing on its main active components, and using modern scientific and technological methods and means to develop traditional Chinese medicine nano-preparations and other pharmaceutical products with independent intellectual property rights, and further develop the unique advantages of improving the hypoxic microenvironment and slowing down joint damage.

In conclusion, the research on the role of hypoxic microenvironment in the occurrence and development of RA has, on a theoretical level, deepened the scientific understanding of the interaction between oxygen, microenvironment, HIF-1α, and multiple receptor expressions and, on the practical level, promoted the research and development of new hypoxic microenvironment regulators. Combining RA drug therapy with hypoxia biomarkers is not only beneficial to the application of hypoxic microenvironment regulation strategy in modern clinical practice but also contributes to the discovery of new drug targets for the treatment of RA and provides scientific prospects for the development of targeted therapy for other immune diseases.

## Glossary

ACPA: Anti-citrullinated protein antibodies
AHR-CRP: Aryl Hydrocarbon Receptor-C-reactive Protein
AKT: Ak Strain Transforming
ANGPTL4: Angiopoietin-like 4
Anti-CarP: Anti-Carbamylated Protein Antibodies
ATP: Adenosine triphosphate
BNIP3: BCL2/Adenovirus e1B 19 kDa Protein-in-teracting Protein 3
C/EBPβ: CCAAT/enhancer binding pro tein 尾
cGAS: Cyclic GMP–AMP synthase
CIA: Collagen-induced arthritis
COX: Cyclooxygenase
CRP: C-reactive protein
CXCL: Chemokine (C-X-C Motif) Ligand
CXCR4: C-X-C chemokine receptor type 4
DCs: Dendritic cells
EMT: Epithelial-mesenchymal transition
EPCs: Endothelial progenitor cells
ERK: Extracellular Signal-regulated Kinase
ESR: Erythrocyte Sedimentation Rate
FIH: Factor inhibiting HIF
FLS: Fibroblast-like synoviocyte
GLUT: Glucose transporters
HBOT: Hyperbaric oxygen therapy
HIF: Hypoxia inducible factor
HMGB-1: High mobility group protein B1
HRE: Hypoxia response element
IFN-γ: Interferon-γ
IL: Interleukin
JAK: Janus-activated kinase
LC-3: Microtubule-Associated Protein 1 Light Chain 3
LDHA: Lactate dehydrogenase
MAPK: Mitogen-activated Protein Kinase
MEK: Mitogen-activated protein kinase kinase
MMP: Metalloproteinases
MTOR: Mammalian Target of Rapamycin
NF-κB: Nuclear Factor Kappa B
NK: Natural Killer Cells
NLRP3: Nod-like Receptor Pyrin Domain Containing 3
Notch-3: Neurogenic Locus Notch Homolog Protein 3
Nrf2: Nuclear Factor-erythroid 2-related Factor-2
OBS: Osteoblast
OCS: Osteoclast
PAD: Peptidylarginine deiminases
PGK1: Phosphoglycerate kinase 1
PHDs: Prolyl hydroxylase domain proteins
PI3K: Phoshatidylinositol-3 Kinase
PTPN22: Protein tyrosine phosphatase non-receptor type 22
RA: Rheumatoid Arthritis
Raf: Extracellular signal-regulated kinase
RANKL: Receptor Activator of Nuclear Factor Kappa-β
ROR γt: Retinoic Acid-related Orphan Receptor Gamma t
ROS: Reactive Oxygen Species
S1P: Sphingosine-1-phosphate
S1PR1: Sphingosine-1-phosphate receptor-1
SDF-1: Stromal cell-derived factor 1
STAT: Signal Transducer and Activator and Transcription
STING: stimulator of interferon genes
TAK1: Transforming growth factor-β-activated kinase 1
Th17: T Helper Cell 17
TLR: Toll-like Receptor
TNF α: Tumor Necrosis Factor Alpha
VEGF: Vascular Endothelial Growth Factor
VHL: Von Hippel-Lindau
Th17: T Helper Cell 17.
